# The Role of Dopamine in Neurological, Psychiatric, and Metabolic Disorders and Cancer: A Complex Web of Interactions

**DOI:** 10.3390/biomedicines13020492

**Published:** 2025-02-17

**Authors:** Luisa Speranza, Maria Concetta Miniaci, Floriana Volpicelli

**Affiliations:** Department of Pharmacy, School of Medicine and Surgery, University of Naples Federico II, 80131 Naples, Italy; luisa.speranza@unina.it (L.S.); maria.miniaci@unina.it (M.C.M.)

Dopamine, a key neurotransmitter in the central nervous system, is essential for regulating a wide range of physiological processes, including motor control, reward processing, mood regulation, and decision-making [1,2,3]. However, its function extends well beyond these traditional roles. Emerging research indicates that dopamine is not only vital for neurological and psychiatric health but also plays a key role in metabolic processes and even cancer progression. The dopamine signaling pathway, comprising the synthesis and release of dopamine, receptor binding, and reuptake, is a complex and finely tuned system that maintains physiological balance and overall well-being. Disruptions within this pathway, however, are linked to various pathological conditions, underscoring its critical role in both health and disease. Altered dopamine signaling is implicated in neurodegenerative disorders like Parkinson’s disease [4], psychiatric conditions such as schizophrenia and addiction [5], and other systemic pathologies. Understanding the molecular mechanisms underlying these disruptions offers opportunities for innovative therapeutic approaches, making it imperative to further investigate dopamine’s role across different biological systems. In this editorial for the Special Issue titled “Dopamine Signaling Pathway in Health and Disease,” we delve into the multifaceted role of dopamine, the consequences of its dysfunction across various physiological systems, and the promising therapeutic potential of modulating its signaling pathways. This investigation is critical for developing more effective and personalized treatments for conditions ranging from Parkinson’s disease (PD) to cancer.

**Dopamine in Parkinson’s Disease**

PD is a neurodegenerative disorder commonly linked to dopamine dysfunction, marked by the gradual loss of dopamine-producing neurons in the midbrain. While the classic motor symptoms of PD—such as tremors, stiffness, and slow movement—are primarily attributed to a lack of dopamine in brain regions like the striatum, recent research has revealed that the underlying molecular mechanisms are much more complex than initially thought [1,4,6].

One of the most compelling research areas is the interaction between dopaminergic receptors, particularly the D1-like (D1R) and D3-like (D3R) receptors, within striatonigral neurons. New findings, such as those from Casados-Delgado and colleagues, emphasize the complexity of these receptor interactions. Their work highlights that dopaminergic denervation disrupts D1R and D3R signaling by altering isoform expression, specifically leading to changes in D3R membrane expression. Importantly, this research identifies a novel mechanistic pathway involving D1R activation and its regulation of D3R splicing through Protein Tyrosine Binding (PTB) phosphorylation. These findings provide a nuanced understanding of how dopamine receptor signaling is finely tuned and how disruptions—such as those induced by D1R blockade or neurodegenerative processes—can impair this balance, potentially contributing to disease pathophysiology [7,8,9].

In this context, there is growing interest in developing selective ligands for dopamine receptors, especially D3R-selective ligands. The goal is to improve drug effectiveness while minimizing side effects. By identifying these selective ligands and understanding their molecular interactions through computational methods like in silico analysis, it is possible to obtain valuable insights into drug design for PD, where the balance between different dopamine receptors, such as the dopamine D2 receptor (D2R) and D3R, is often disrupted [10,11].

In addition to motor symptoms, deficits in cognitive flexibility have also been identified in PD. Recent findings suggest that cognitive flexibility deficits in Parkinson’s disease (PD) are linked to dysfunction in a dopamine-modulated cortical–striatal circuit, particularly involving the medial prefrontal cortex (mpFC) and nucleus accumbens (NAc). These regions are crucial for processing the motivational value of stimuli, which is often disrupted in PD. Changes in dopaminergic transmission within these circuits can contribute to the alteration of cognitive flexibility and exacerbate non-motor symptoms of PD. Experimental studies, including those on Pavlovian extinction, support the idea that dopamine levels may increase in the mpFC. At the same time, dopaminergic transmission is inhibited in the NAc, shedding light on potential mechanisms underlying PD and other neurodegenerative disorders [12].

Moreover, the intricate interplay between dopamine and serotonin systems adds another layer of complexity to the development of dopamine-based therapies. Research by Traktirov in rats shows that knockout of the dopamine transporter (DAT) gene, which regulates dopamine reuptake into presynaptic neurons, impacts dopamine signaling and serotonin levels across multiple brain regions. Changes in DAT lead to widespread disruptions in neurotransmission and altered mRNA expression associated with monoamine metabolism enzymes like MAO-A, MAO-B, and COMT. This highlights that interventions targeting dopamine pathways could inadvertently affect serotonin systems, suggesting a need for nuanced, multi-targeted approaches [13,14].

Epigenetic inheritance also offers critical insights into neuropsychiatric and neurodegenerative vulnerabilities. Recent studies on DAT knockout (DAT-KO) and heterozygous (DAT-HET) rats [15] have revealed that genetic and epigenetic modifications of the DAT gene can introduce further complexities in dopamine and serotonin interactions. DAT-KO rats display stereotypical behaviors, hyperactivity, and deficits in cognitive function, with altered circadian rhythms and working memory. Interestingly, the epigenetic modulation of the DAT gene through paternal lineage (specifically, the QULL model) has shown that the paternal side’s uterine environment can influence behavior across generations. Offspring from a MAT-HET dam × QULL sire cross exhibited compulsive and obsessive behaviors, even though all rats were heterozygous. These results indicate that the transgenerational epigenetic inheritance of dopamine-related traits could significantly impact behavior, especially considering the combined effects of the dopamine and serotonin systems [16,17].

Neuroinflammation has emerged as a significant contributor to dopaminergic neurodegeneration, particularly in Parkinson’s disease, highlighting the role of activated microglia in exacerbating midbrain dopaminergic neuron loss through the release of neurotoxic factors. Toxins such as 2,4-dichlorophenoxyacetic acid (2,4D), an herbicide commonly used in agriculture, have been shown to exacerbate dopaminergic neuron loss by triggering microglia-mediated neuroinflammation. Research led by Russ and coworkers has revealed that while 2,4D does not directly activate microglia, its neurotoxic effects are markedly enhanced in their presence. Specifically, exposure to 2,4D in neuron–glia cultures correlates with increased TNFα release—an inflammatory cytokine strongly linked to neurotoxicity. This points to microglial reactivity as a key mechanism by which neuroinflammation accelerates neuron death in Parkinson’s disease. These findings suggest that environmental exposures may interact with genetic predispositions to increase the risk of developing PD, underscoring the importance of considering genetic and environmental factors in the disease’s progression. The interaction between environmental toxins and dopamine dysfunction further highlights the need for a multifaceted approach to PD prevention and treatment [18,19].

**Dopamine in Neuropsychiatric Disorders**

In psychiatric disorders such as schizophrenia, dopamine plays a pivotal role in modulating mood, cognition, and behavior. The symptoms of schizophrenia, which include hallucinations, delusions, and cognitive impairment, are often attributed to dysregulated dopamine activity in the brain [20,21]. Antipsychotic medications, which primarily block dopamine D2 receptors, are the cornerstone of treatment for many patients. However, a subset of patients with treatment-resistant schizophrenia (TRS) fails to respond adequately to these medications, posing a significant challenge to clinical practice.

Recent research suggests that the mechanisms behind TRS are multifactorial, involving abnormalities in dopamine receptor function, presynaptic dopamine synthesis and release, and postsynaptic neurotransmission. One contributing factor may be an increased density of D2 receptors in the brain, particularly in the high-affinity state, which could limit the effectiveness of D2 receptor-blocking antipsychotic medications [22,23]. Moreover, dopamine receptor heteromers—complexes formed by different dopamine receptor subtypes—may play a role in the pathophysiology of schizophrenia, further challenging the treatment interventions.

Cardiotonic steroids, which act as ligands for Na^+^ and K^+^-ATPase, are emerging as significant players in both physiological and pathological processes. However, their role in the central nervous system is still not fully understood. Evidence suggests that endogenous cardiotonic steroids are implicated in mood disorders, such as depression and bipolar disorders, which are linked to dysfunctions in the dopaminergic system. Animal studies have demonstrated that the cardiotonic steroid ouabain induces mania-like behavior through dopamine-dependent intracellular signaling pathways. Additionally, alterations in Na^+^ and K^+^-ATPase expression directly affect dopaminergic systems and can be implicated in neurodegenerative diseases [24]. Thus, understanding the role of cardiotonic steroids and Na^+^, K^+^-ATPase in dopaminergic dysfunctions is essential for developing targeted treatments for these complex disorders.

**Dopamine and Addiction: A Disruption of Reward Pathways**

Dopamine plays a central role in regulating the mesolimbic system, also known as the brain’s reward system. This system governs behaviors and emotional responses related to motivation, pleasure, and the pursuit of rewards.

The process of addiction involves the rewiring of reward circuits, where repeated exposure to addictive substances like drugs, alcohol, or even certain behavioral stimuli can hijack the brain’s reward system, leading to altered patterns of reinforcement and behavior. Functional changes in dopamine pathways, exacerbated by environmental stressors like the COVID-19 pandemic, have contributed to maladaptive reward-seeking behaviors. During lockdown periods, feelings of isolation and heightened stress caused many individuals to turn to food or digital rewards as coping mechanisms. This reliance disrupted dopamine-driven reward systems, increasing the risk of compulsive behaviors, sleep disturbance, depression, and suicidal thoughts [6].

Research into opioid use disorder (UOD) and methamphetamine’s (METH) effects on cognition has revealed important neurobiological pathways and potential treatment targets. Methamphetamine disrupts dopamine signaling, affecting neuroplasticity and cognitive function. Reduced brain-derived neurotrophic factor (BDNF), essential for neural plasticity, exacerbates these effects [25]. Animal studies reveal that mice with lower BDNF expression exhibit deficits in prepulse inhibition (PPI), a marker of psychosis. A recent study found that male mice with reduced BDNF expression showed more pronounced PPI disruptions following chronic methamphetamine exposure, independent of dopamine D3 receptor activity. In contrast, female mice showed no such effects, highlighting sex-specific differences in how BDNF modulates methamphetamine’s impact on cognition [26].

Similarly, research on opioid use disorder (OUD) has found sex differences in opioid-induced cognitive impairment. Men with OUD exhibit altered event-related potentials (ERPs) and longer latencies in cognitive tasks, while women with OUD show shorter latencies and slower reaction times. These findings suggest that both methamphetamine and opioid addiction differentially affect cognitive processing in men and women [27,28]. Taken together, these studies underscore the role of BDNF in addiction-related cognitive deficits and suggest that targeting both dopamine and BDNF could improve therapeutic outcomes for addiction and related neuropsychiatric disorders [26].

**Dopamine in Metabolic Disorders and Cancer**

Dopamine’s influence is not confined to the brain. Increasing evidence suggests that it is essential in regulating metabolic processes, including glucose metabolism and insulin sensitivity. In conditions such as type 2 diabetes, dopamine dysregulation has been implicated in both insulin resistance and cognitive decline, indicating that neurological and metabolic health are closely intertwined. This dual role of dopamine suggests that therapies targeting dopaminergic pathways could have wide-ranging benefits, addressing the metabolic and cognitive dysfunctions associated with diseases like diabetes [29,30].

Additionally, dopamine is involved in bone remodeling, influencing osteoclast activity, and is essential for maintaining bone density. Chronic use of psychostimulants such as methylphenidate and amphetamine, which are commonly prescribed for attention-deficit hyperactivity disorder (ADHD), can disrupt dopamine signaling and negatively affect bone health [31]. These drugs have increased osteoclast activity, reducing bone density and structural integrity. This unexpected consequence highlights the importance of monitoring the systemic effects of dopaminergic medications, especially when used over long-term [32].

Dopamine is also implicated in tumor biology, with evidence suggesting its influence on cancer development, progression, and response to treatment [33,34,35]. Its role in cancer involves multiple pathways, including cell proliferation, angiogenesis, metastasis, and immune response. Some existing drugs initially developed for non-oncological purposes have been explored for their potential in cancer treatment due to their interaction with dopamine receptors. These drugs can act as antagonists, inhibiting tumor cell growth and promoting cell death. Repurposing these drugs offers promising therapeutic opportunities, although more research is needed to clarify the mechanisms involved and determine these approaches’ clinical efficacy and safety [36].

**Conclusions**

Dopamine’s physiological significance extends well beyond its classical roles in mood regulation, reward processing, and motor control. Its intricate interaction with diverse biological systems underscores its involvement in various critical processes, including neurological, psychiatric, metabolic, and oncological disorders [37]. Dopamine’s multifaceted role highlights its potential as a therapeutic target and the complexity of its regulatory mechanisms in disease pathogenesis [5]. In Parkinson’s disease, dopamine dysfunction contributes to motor and non-motor symptoms, highlighting the need for advanced therapeutic strategies targeting dopaminergic pathways. Similarly, in psychiatric disorders such as schizophrenia [38] and addiction, dysregulated dopamine signaling not only affects cognition and behavior but also complicates treatment strategies, particularly in treatment-resistant cases. Furthermore, genetic predispositions and environmental influences are increasingly recognized as critical modulators of dopaminergic pathways, further complicating the development of effective and individual treatment.

Moreover, dopamine’s influence is increasingly recognized outside the brain. It impacts metabolic processes such as glucose regulation and insulin sensitivity, which are implicated in the pathogenesis of metabolic diseases like type 2 diabetes. These findings suggest that therapies targeting dopamine could offer dual benefits, addressing neurological and metabolic dysfunctions [39]. In the cancer context, exploring dopamine signaling pathways offers exciting prospects. Research into dopamine receptor modulators has demonstrated the potential for inhibiting tumor growth, suggesting opportunities for repurposing existing dopamine-targeting drugs as novel cancer therapies. These insights represent a promising translational application of dopamine research with the potential to alter standard oncological treatment paradigms.

As we unravel dopamine’s multifaceted roles, it becomes clear that a more integrated approach is needed to develop effective, personalized treatments for the wide range of disorders influenced by this essential neurotransmitter. Future research on the nuanced interactions between dopamine and other systems will be critical for advancing our understanding and therapeutic capabilities in treating these conditions, which range from neurodegenerative and psychiatric disorders to metabolic and oncological diseases.
